# Book Review: Memory: A History

**DOI:** 10.3389/fpsyg.2015.02047

**Published:** 2016-01-12

**Authors:** Kourken Michaelian

**Affiliations:** Department of Philosophy, University of OtagoDunedin, New Zealand

**Keywords:** memory, history of philosophy, history of psychology, theoretical psychology, collective memory

As the first to provide a thorough overview of the history of philosophical thinking about memory, this book fills an important gap in the literature (Nikulin, 2015). Despite some minor omissions in its coverage and some unusual features of its format (see below), Dmitri Nikulin's edited collection does an admirable job of providing a synoptic view of memory in the history of philosophy, and it will constitute a valuable addition to the libraries not only of philosophers but also of psychologists interested in the development of a concept which continues to play a central role in psychological research. Due to the length and scope of the multi-author volume—the book contains eight chapters on different aspects of memory in the history of philosophy, as well as a substantial editorial introduction and a series of brief “reflections” intended to relate philosophical themes to ideas from other fields—only a cursory description of its contents can be provided here; in particular, the “reflections” will be discussed only in passing.

Nikulin's chapter on memory in *ancient philosophy* provides detailed accounts of memory and recollection in Plato and Aristotle, both of which exerted a major influence on subsequent philosophers, but the chapter goes well beyond these figures to discuss memory in the Stoics, Roman thought, and Plotinus. Jörn Müller's chapter on memory in *medieval philosophy* is similarly innovative, focusing on explicit theories of memory—including those proposed by Augustine, Avicenna, Averroes, Albert the Great, and Thomas Aquinas—rather than the practices or arts of memory that usually take centre stage in accounts of medieval views of memory. Both chapters will serve to introduce readers to some often-overlooked theoretical resources.

Stephen Clucas's chapter on memory in *renaissance and early modern philosophy* traces the shift from a broadly Aristotelian view of memory as a distinct faculty to a view of memory as one function of a unified intellect, discussing the blurring of the boundary between memory and imagination in figures including Descartes and Hobbes. The chapter also traces the gradual unlinking of memory and knowledge during this period and the linking of memory and personal identity in Locke. Given the continuing influence of many of the figures discussed here, the chapter will be a particularly valuable reference. Angelica Nuzzo's chapter on memory in *classical German philosophy* is considerably more technical and may be somewhat challenging for readers unfamiliar with the period. This was likely inevitable, given that many of the figures discussed — from Kant through to Hegel — presupposed views of memory but did not provide systematic treatments of the topic, and the chapter nevertheless provides a useful resource for contemporary analytic philosophy of memory, which tends not to make much reference to philosophers from this period.

Nicolas de Warren's chapter on memory in *continental philosophy* is similarly challenging, though for different reasons. Many of the thinkers discussed here did provide explicit treatments of memory, but each had his own problematic and employed his own technical vocabulary. Despite this, the chapter provides a clear overview of themes as diverse as habit memory (in Bergson), primary vs. secondary memory in relation to time-consciousness (in Husserl), and the “forgetting of being” (in Heidegger). Turning from continental to *analytic philosophy*, Sven Bernecker's chapter reviews contemporary controversies over distinctions among kinds of memory and epistemic vs. causal theories of memory before focusing on the epistemology of memory, including the possibility of memory without belief and without justification, scepticism about memory knowledge, and generationism vs. preservationism. The chapter concludes with a helpful discussion of contemporary treatments of the relationship between memory and personal identity.

The remaining two chapters—on *memory and culture*, by Jan Assmann, and on *trauma, memory, and holocaust*, by Michael Rothberg—have a thematic rather than a historical focus. In view of the increasing importance of accounts of collective memory, the inclusion of Assman's chapter gestures usefully toward the need for work linking traditional philosophical treatments of memory to contemporary debates over collective memory in the social sciences. In contrast, Rothberg's chapter, as the only chapter to focus on memory for a particular event rather than general theories of memory, to some extent fails to connect with the rest of the volume.

Any volume as ambitious in its scope as this one inevitably includes multiple claims or arguments that invite disagreement, but the authors collectively do an excellent job of surveying a broad range of philosophical thinking about memory. The only major limitations of the volume pertain to some omissions in its coverage and some features of its format. As far as content is concerned, Hume does not make much of an appearance, despite the importance of his treatment of the relationship between memory and imagination. And there is little discussion of the emergence of empirical approaches (such as behaviorism) to memory in psychology, potentially limiting the usefulness of the book to readers outside of philosophy. Along the same lines, the book does not discuss contemporary research, inspired by distributed cognition and similar theoretical frameworks, on offloading to external memory; while links could have been drawn between this research and, for example, Plato's arguments on the effects of writing on memory, it might have been difficult to find sufficient space in the volume. And the book does manage to find space for a contemporary cognitive neuroscience perspective, in the form of a brief “reflection” on memory as an adaptive constructive process by Daniel Schacter. As far as format is concerned, however, the inclusion of the “reflections” (required by the format of the Oxford Philosophical Concepts series) sometimes gives the book a disjointed feel; for example, a brief “reflection” on memory in daoism is sandwiched between the lengthy chapters on memory in the renaissance and early modern philosophy and memory in classical German philosophy. To the extent that they permit coverage of topics that otherwise could not have been discussed, however, the reflections are helpful on the whole.

These criticisms are minor. The book was an extremely ambitious project, and the editor and authors are to be applauded for having carried it out successfully. Philosophers owe them a debt of gratitude for providing a comprehensive overview of the history of the philosophy of memory. And psychologists will benefit from the availability of a detailed account of the historical origins of a concept which remains fundamental for their research today.

## Author contributions

KM is the sole author of the review.

### Conflict of interest statement

The author declares that the research was conducted in the absence of any commercial or financial relationships that could be construed as a potential conflict of interest.
